# Extracellular vesicles derived from the lactic acid bacterium *Enterococcus rotai* suppress melanogenesis by modulating the cAMP-MITF pathway

**DOI:** 10.1186/s40643-026-01121-5

**Published:** 2026-09-12

**Authors:** Yunsik Kim, Jin Hee Lee, Eun-Gyung Cho

**Affiliations:** 1 Extracellular Vesicles and Biomaterials Center, CHA Research Institute, CHA Bundang Medical Center, CHA University School of Medicine, Seongnam, 13496 Republic of Korea; 2 Department of Biochemistry, CHA University School of Medicine, Pocheon, 11160 Republic of Korea

**Keywords:** Extracellular vesicles, *Enterococcus rotai* CMTB-CA6, *Centella asiatica*, Anti-melanogenesis, Phosphodiesterase activity, cAMP suppression, Antioxidant

## Abstract

*Enterococcus rotai* CMTB-CA6-derived extracellular vesicles (CA6-ErEVs), isolated from a *Centella asiatica*-associated lactic acid bacterium, were investigated for their potential to modulate oxidative stress, inflammation, and melanogenesis in skin cells. CA6-ErEVs were purified and characterized using standardized nanoparticle analyses. Functional assays demonstrated that CA6-ErEVs displayed robust antioxidant activity by reducing free radical accumulation and intracellular reactive oxygen species levels. In tumor necrosis factor-α–stimulated dermal fibroblasts, CA6-ErEVs attenuated the secretion of proinflammatory cytokines and reduced matrix degradation while promoting procollagen synthesis. In α-melanocyte–stimulating hormone–activated murine and human melanocytes, CA6-ErEVs markedly suppressed melanin synthesis and tyrosinase activity, accompanied by the downregulation of microphthalmia-associated transcription factor (MITF) and key melanogenic enzymes including tyrosinase, tyrosinase-related protein 1, and tyrosinase-related protein 2. Mechanistically, CA6-ErEVs reduced intracellular cyclic adenosine monophosphate (cAMP) levels through intrinsic phosphodiesterase activity, thereby inhibiting the cAMP-MITF signaling axis and oxidative pathways involved in melanogenesis. Collectively, CA6-ErEVs exhibit integrated antioxidant, anti-inflammatory, and antimelanogenic effects through phosphodiesterase-mediated suppression of cAMP signaling and regulation of cellular redox balance. These findings highlight bacterial EVs derived from *E. rotai* as promising bioactive candidates for further development in the management of hyperpigmentation and improving cutaneous homeostasis.

**Graphical abstract:**

**Figure:**
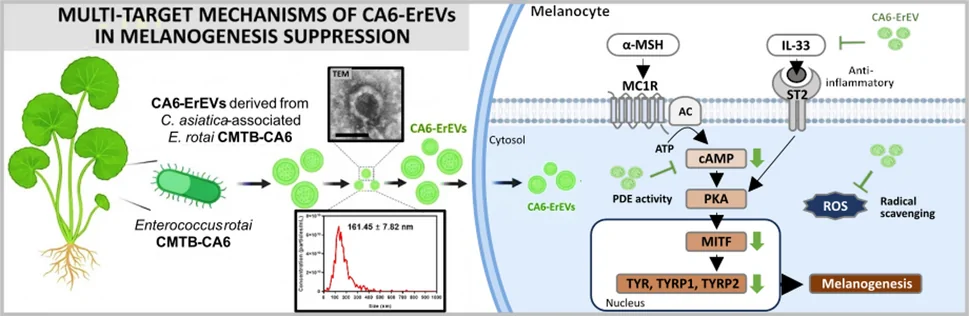

**Supplementary information:**

The online version contains supplementary material available at https://doi.org/10.1186/s40643-026-01121-5.

## Introduction

Extracellular vesicles (EVs) are typically < 200 nm in diameter and composed of membrane-bound sacs formed by phospholipid bilayers (Tenchov et al. 2022). EVs are released by all cellular organisms from unicellular microbes to complex mammals (Rybak and Robatzek 2019). Because EVs contain biomolecules derived from their parent cells, they play a crucial role in intercellular communication (Li et al. 2024). This communication extends beyond intercellular interactions and includes cross-kingdom communication. EVs facilitate communication between cells and alter the function of recipient cells, thereby contributing to processes such as tissue repair, immune responses, and disease progression (Ragni 2025).

Probiotics, primarily lactic acid bacteria (LAB), provide numerous health benefits to humans (Latif et al. 2023; Gul and Durante-Mangoni 2024). LAB exert diverse effects on the skin, including moisturizing, antioxidant, anti-inflammatory, and microbiome-modulating activities (Lee et al. 2023; Prajapati et al. 2025; Song et al. 2026; Zhao et al. 2022). EVs derived from LAB resemble naturally occurring phospholipid nanoparticles and can be efficiently absorbed, enabling penetration through the skin barrier (Lee et al. 2023; Sun et al. 2025). Compared with bacterial cell lysates, LAB-derived EVs exhibit lower cytotoxicity and higher bioactivity even at lower concentrations.

Plant-derived bioactive compounds have been extensively documented to exert beneficial dermatological effects, including antioxidant and anti-inflammatory properties, thereby contributing to overall cutaneous homeostasis (Diniz et al. 2023; Seo et al. 2025; Tu et al. 2025). Building on these botanical benefits, Plant-associated LAB represent a particularly promising source for skincare applications. These strains produce diverse secondary metabolites that enable adaptation to harsh environments and exhibit strong tolerance to salinity, acidity, and bile (Cho et al. 2009). Among medicinal plants, *Centella asiatica* (Gotu Kola), long recognized for its wound healing properties and efficacy against acne, psoriasis, eczema, and dermatitis due to bioactive compounds such as asiaticoside, madecassoside, asiatic acid, and madecassic acid (Park 2021; Diniz et al. 2023), serves as a source of plant-associated LAB.

Despite its potent antimicrobial nature potentially limiting microbial investigation (Biswas et al. 2021; Bandopadhyay et al. 2023), in our previous study, we identified *Enterococcus rotai* CMTB-CA6, from *C. asiatica* and demonstrated its antimicrobial, anti-inflammatory, and antioxidant properties−even when cultured in standard LAB medium without *C. asiatica* supplementation (Kim et al. 2024).

While LAB-derived EVs demonstrate antioxidant and anti-inflammatory properties, their potential to regulate melanogenesis−particularly in the context of inflammation-associated hyperpigmentation−remains underexplored. Moreover, the bioactivities of EVs derived from plant-associated LAB, such as those isolated from *C. asiatica*, have not yet been systematically characterized in skin cells. Here we investigated EVs from *E. rotai* CMTB-CA6 (CA6-ErEVs) to elucidate their integrated effects on oxidative stress, inflammatory responses, and pigmentation pathways in human dermal fibroblasts (HDFs) and melanocytes. These findings characterize the therapeutic potential of plant-associated LAB-derived EVs for dermatological applications.

## Results

### Isolation of EVs from *E. rotai* CMTB-CA6 (CA6-ErEVs)

**Fig. 1:**
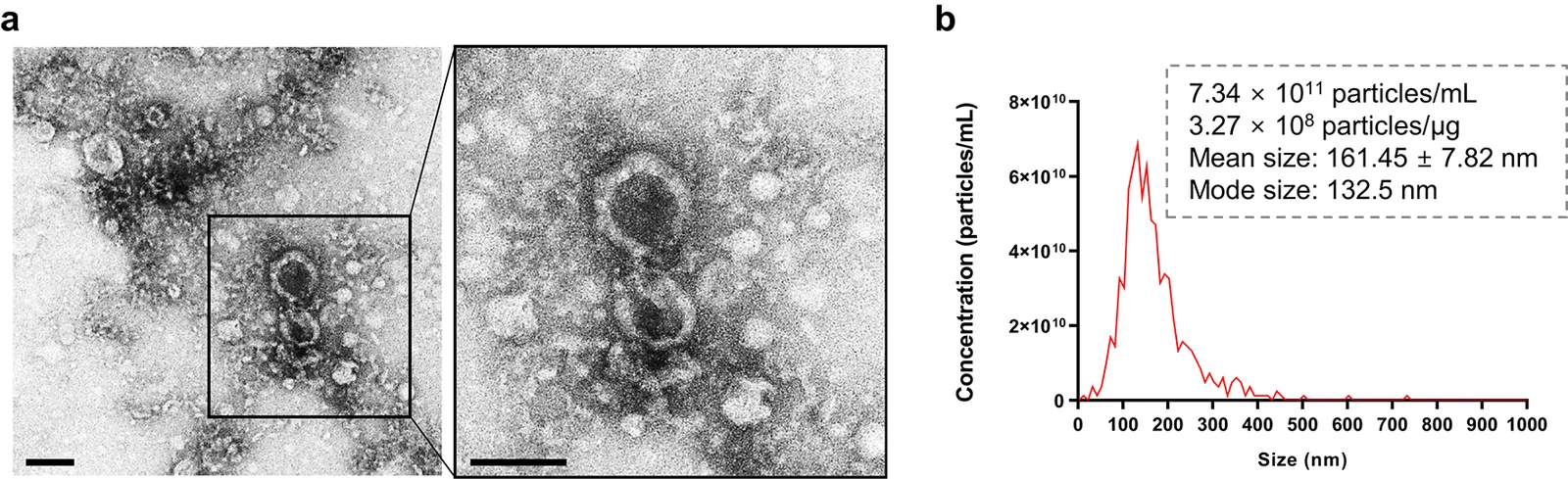
Characterization of extracellular vesicles isolated from *Enterococcus rotai* CMTB-CA6 (CA6-ErEVs). **a** Representative bio-transmission electron microscopy (TEM) images of purified CA6-ErEVs are shown. Scale bars, 100 nm. **b** Particle size distribution and concentration of CA6-ErEVs were determined using nanoparticle tracking analysis (NTA), with representative data presented

Extracellular vesicles from *E. rotai* CMTB-CA6 (CA6-ErEVs) were purified and concentrated using a combination of tangential flow filtration and ultracentrifugation to characterize their physical properties and investigate their beneficial effects on human skin. Biological transmission electron microscopy (Bio-TEM) analysis revealed that CA6-ErEVs had a closed spherical membrane structure (Fig. 1a). Nanoparticle tracking analysis (NTA) showed that CA6-ErEVs had a mean diameter of 161.45 ± 7.82 nm (mode diameter, 132.5 nm) and a particle-to-protein ratio of 3.27 × 10⁸ particles/μg (Fig. 1b). SDS-PAGE analysis further demonstrated that the protein profile of CA6-ErEVs was clearly distinct from that of the parental whole lysate (CA6-WL), indicating selective enrichment of EV-associated protein cargo (Supplementary Figure S1a). Total nucleic acids associated with CA6-ErEVs were quantified following TRIzol extraction (Supplementary Figure S1b). The DNA/RNA ratio was 1:7.09 in CA6-ErEVs, compared with 1:1.49 in CA6-WL, demonstrating that RNA constituted the predominant nucleic acid component in CA6-ErEVs and that the nucleic acid composition differed markedly between CA6-ErEVs and CA6-WL. To further assess EV purity, OptiPrep density gradient ultracentrifugation was performed. CA6-ErEVs were predominantly recovered at a density of 1.12–1.14 g/cm^3^ (Supplementary Figure S1c). Following OptiPrep purification, the vesicles retained a spherical morphology (Fig. 1a), and the mode diameter (122.5 nm) remained comparable to that of the bulk TFF-purified preparation (132.5 nm). In addition, the particle-to-protein ratio increased from 3.27 × 10⁸ to 9.32 × 10⁸ particles/μg after OptiPrep purification. Because the protein content remained largely unchanged after OptiPrep purification, these results suggest that the large-scale TFF purification process produced CA6-ErEVs with minimal contamination by non-vesicular proteins. These findings confirmed that CA6-ErEVs exhibited the characteristic morphology, size distribution, and density profile of LAB-derived EVs, with a high particle-to-protein ratio, supporting their successful isolation and purification.

### Enhanced antioxidant capacity of CA6-ErEVs compared with CMTB-CA6

**Fig. 2:**
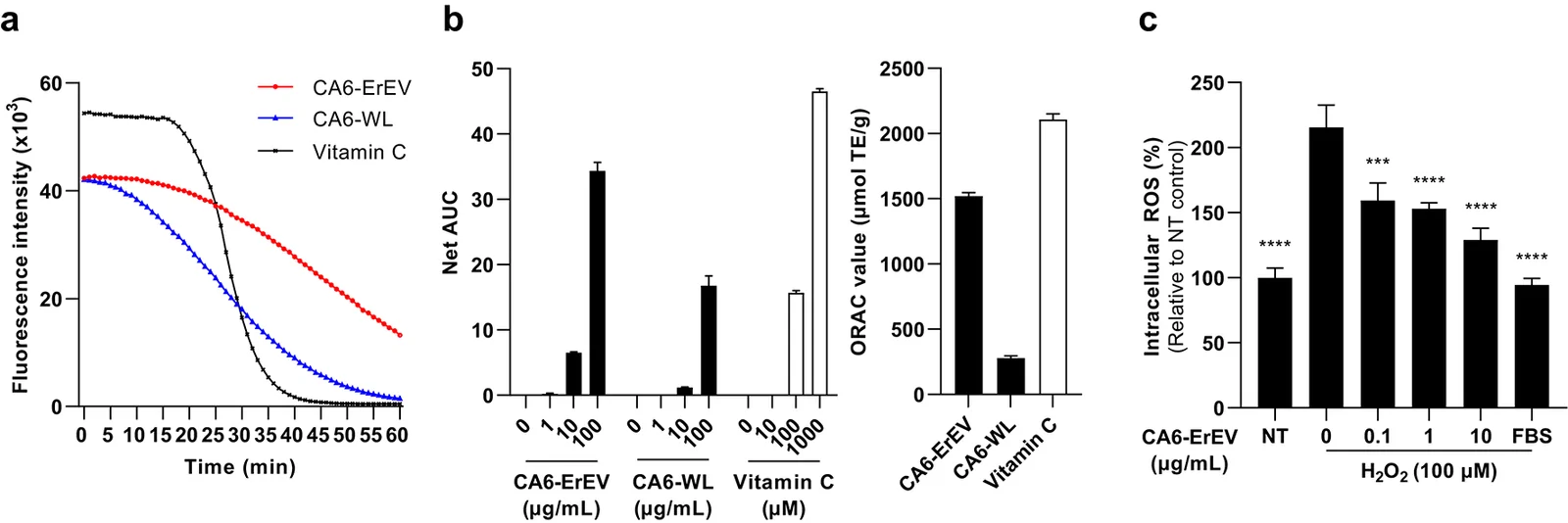
Antioxidant effects of CA6-ErEVs. **a** Oxygen radical absorbance capacity (ORAC) assay was used to assess antioxidant activity of CA6-ErEVs and whole lysate of CMTB-CA6 (CA6-WL) at 100 μg/mL, with fluorescence intensity monitored over time (Vitamin C served as a positive control). **b** Net area under the curve (AUC) was calculated as AUC_sample − AUC_blank. Antioxidant capacity was quantitatively expressed as Trolox equivalents (μmol TE/g) using a standard curve (R^2^ > 0.98); Vitamin C was used as positive control (white bars). (**c**) Intracellular reactive oxygen species (ROS) levels were measured in HaCaT cells using CM-H_2_DCFDA probe and expressed as percentage of untreated control (NT). Data represent mean ± standard deviation (SD) (n = 3); statistical significance was determined using one-way analysis of variance (ANOVA) versus H_2_O_2_ alone. *** *p* < 0.001, **** *p* < 0.0001. NT, no treatment

Bacterial lysates of* E. rotai* CMTB-CA6 (CA6-WL) exert beneficial effects on the human skin, including antioxidant and anti-inflammatory activities (Kim et al. 2024). We performed an oxygen radical absorbance capacity (ORAC) assay to compare the antioxidant capacities of CA6-ErEVs and CA6-WL. This method measures the antioxidant capacity of reactive oxygen species (ROS)-scavenging agents by monitoring the time-dependent decay of fluorescent signals, where a slower decay indicates a stronger antioxidant effect. At 100 µg/mL, CA6-ErEVs delayed fluorescence decay more effectively than CA6-WL, exhibiting a decay profile comparable to that of the positive control, vitamin C (Vc) (Fig. 2a). Consistent with this finding, the net AUC of CA6-ErEVs at 100 µg/mL was 34.3, which was significantly higher than that of CA6-WL (16.8) (Fig. 2b). CA6-ErEVs also exhibited higher net AUC values than CA6-WL at all tested concentrations (1, 10, and 100 µg/mL), demonstrating their superior antioxidant capacity. Based on the Trolox standard curve, the antioxidant capacity of CA6-ErEVs was calculated to be 1,518.95 µmol TE/g, corresponding to 544.4% of that of CA6-WL and reaching 72.1% of the level observed with Vc (2,107.8 µmol TE/g) (Fig. 2b). We next investigated whether this antioxidant activity was also evident at the cellular level by assessing the effects of CA6-ErEVs on H₂O₂-induced intracellular ROS accumulation in HaCaT cells. Exposure to H₂O₂ markedly increased intracellular ROS levels, whereas treatment with CA6-ErEVs dose-dependently suppressed H₂O₂-induced ROS accumulation (Fig. 2c). These findings demonstrate that the antioxidant capacity of CA6-ErEVs observed in the ORAC assay was retained at the cellular level, thereby protecting human keratinocytes against oxidative stress.

### Anti-inflammatory effects of CA6-ErEVs on tumor necrosis factor (TNF)-α-treated HDFs

**Fig. 3:**
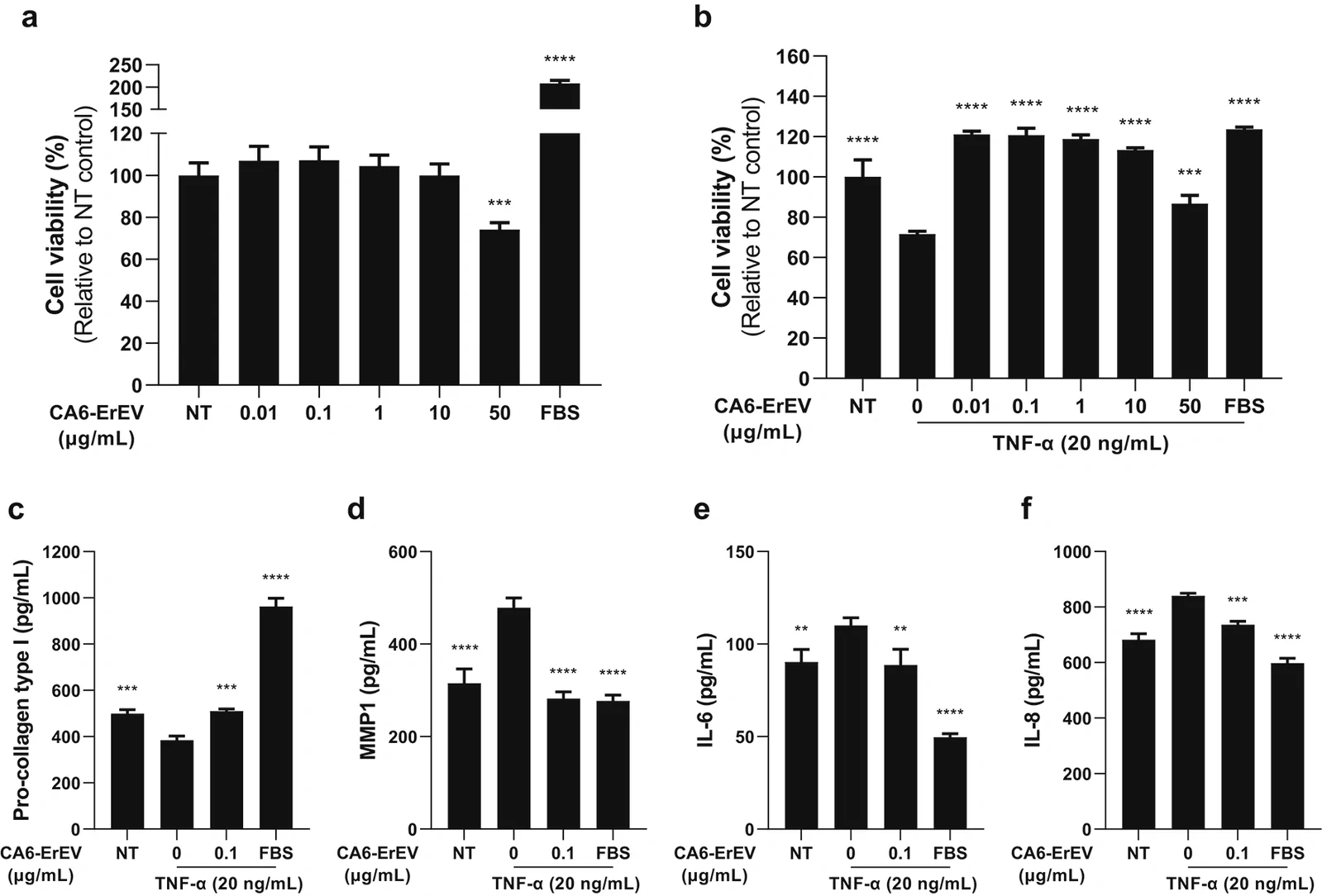
Effects of CA6-ErEVs on tumor necrosis factor-α (TNF-α)-treated human dermal fibroblasts (HDFs). **a** HDFs were treated with CA6-ErEVs (0.01–50 μg/mL) for 24 h, and cell viability was assessed using cell counting kit-8 (CCK-8) assay. **b** HDFs were serum-starved for 6 h, treated with 20 ng/mL TNF-α for 24 h in serum-free conditions, followed by CA6-ErEVs (0.01–50 μg/mL) for an additional 24 h. Cell viability was determined using CCK-8 assay. **c**–**f** Conditioned media were collected 24 h post CA6-ErEVs (0.1 μg/mL) treatment under TNF-α conditions. Pro-collagen type I, matrix metalloproteinase−1 (MMP-1), interleukin-6 (IL-6), and IL-8 levels were quantified using specific enzyme-linked immunosorbent assay (ELISA) kits. Data represent mean ± SD (n = 3). For (**a**), statistical analysis by one-way ANOVA versus NT control; for (**b**–**f**), versus TNF-α alone. ** *p* < 0.01, *** *p* < 0.001, **** *p* < 0.0001. NT, no treatment; SF, serum-free control; FBS, 10% fetal bovine serum served as a furcntioanl postive cotnrol; TNF-α, tumor necrosis factor-α

Both *C. asiatica* and *E. rotai* CMTB-CA6 exhibit anti-inflammatory effects (Kim et al. 2024). Hence, CA6-ErEVs were hypothesized to possess anti-inflammatory properties. Before assessing their anti-inflammatory activity, we evaluated the cytotoxicity of CA6-ErEVs in HDFs. CA6-ErEVs showed no cytotoxicity toward HDFs at a concentration range of 0.01–10 μg/mL (Fig. 3a). TNF-α is a major inflammatory cytokine in the human skin, amplifying proinflammatory cytokine levels through the inflammatory signaling cascade (Pocino et al. 2024), contributing to matrix-degrading and inflammatory phenotypes (Banno et al. 2004; Pocino et al. 2024). Therefore, HDFs were stimulated with TNF-α to induce an inflammatory condition and matrix-degrading phenotype, followed by treatment with CA6-ErEVs to test whether CA6-ErEVs could recover TNF-α-induced cellular phenomena. The cell viability of TNF-α-treated HDFs significantly decreased compared with those of the serum-free control (Fig. 3b). Treatment with various concentrations of CA6-ErEVs significantly recovered cell viability (Fig. 3b), suggesting that CA6-ErEVs restored TNF-α-induced inflammatory phenotypes in HDFs. Because CA6-ErEVs were effective at low concentrations without cytotoxicity, 0.1 μg/mL was selected for subsequent experiments.

TNF-α-treated HDFs exhibited inhibited collagen synthesis, increased matrix metallopeptidases (MMPs) levels, and upregulated proinflammatory cytokines, including interleukin-6 (IL-6) and IL-8 (Lee et al. 2023). As such, TNF-α treatment reduced procollagen type I levels; however, CA6-ErEV treatment recovered collagen synthesis to untreated control levels (Fig. 3c). In contrast, the TNF-α-induced upregulated levels of MMP1, a secreted collagenase, were repressed by CA6-ErEVs (Fig. 3d). The levels of senescence-associated inflammatory cytokines IL-6 and IL-8 were considerably downregulated by CA6-ErEVs treatment (Fig. 3e–f). These results demonstrated that CA6-ErEVs can mitigate skin inflammation and matrix-degrading phenotypes in TNF-α-challenged HDFs.

### CA6-ErEVs inhibit melanin production in mouse melanoma cells and human epidermal melanocytes

Extracts of *C. asiatica* influence melanocyte activity, exerting skin-whitening effects (Idana et al. 2022; Seo et al. 2025). However, no evidence exists for *C. asiatica*-associated LAB regulating melanogenesis or skin pigmentation.

**Fig. 4:**
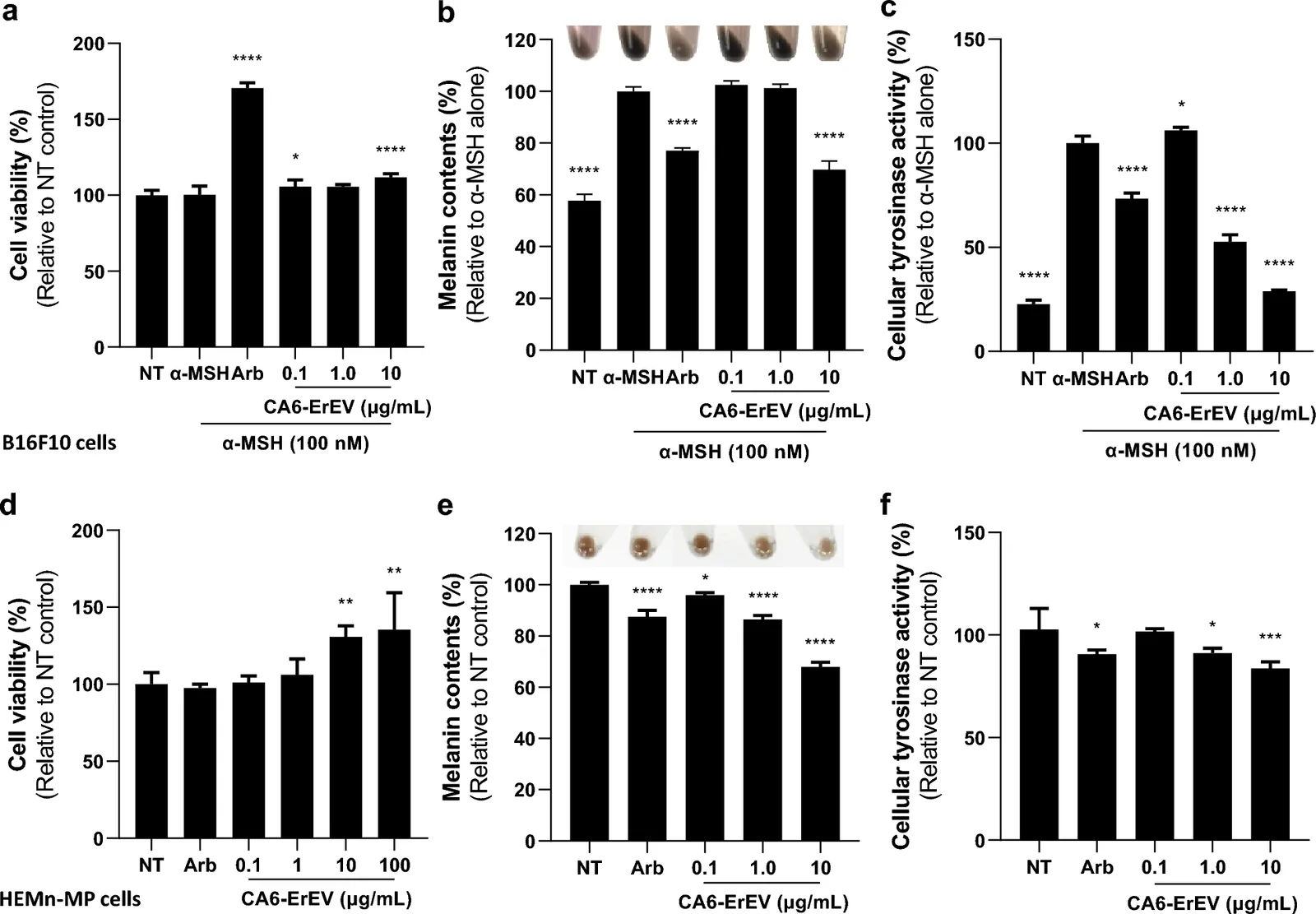
Anti-melanogenic effects of CA6-ErEVs in B16F10 and human epidermal melanocytes (HEMs). **a, d** Cell viability was assessed using CCK-8 assay in B16F10 (**a**) and HEMs (**d**) following treatment with various concentrations of CA6-ErEVs and arbutin (Arb, 100 μg/mL). **b, e** Melanin contents were quantified using melanin assay in α-melanocyte–stimulating hormone (α-MSH)–stimulated B16F10 (**b**) and HEMs (**e**) treated with CA6-ErEVs (0.1, 1, 10 μg/mL); 100 nM α-MSH served as inducer and 100 μg/mL Arb as positive control. Data were normalized to the total protein content determined by a BCA assay. **c, f** Cellular tyrosinase activity was measured in α-MSH–stimulated B16F10 (**c**) and HEMs (**f**) following CA6-ErEVs and Arb treatment. Data represent mean ± SD (n = 4; one-way ANOVA). Panels (**a**, **d**–**f**) compared to untreated (NT) control; panels (**b**–**c**) compared to α-MSH alone. * *p* < 0.05, ** *p* < 0.01, *** *p* < 0.001, **** *p* < 0.0001. Arbutin (Arb; 100 μg/mL), a standardized reference agent for melanogenesis inhibition, was used as the positive control. α-MSH, α-melanocyte–stimulating hormone

We treated murine B16F10 melanoma cells with α-melanocyte–stimulating hormone (α-MSH) to induce melanogenesis. Cotreatment with α-MSH and CA6-ErEVs slightly increased B16F10 cell viability compared with that of the untreated control (Fig. 4a). Melanin production was stimulated by 100 nM α-MSH; however, treatment with 10 μg/mL CA6-ErEVs significantly reduced the α-MSH-induced melanin content by 30.24%, whereas 100 μg/mL arbutin (Arb), a known inhibitor of melanin synthesis, reduced it by 22.90% (Fig. 4b). The melanin-reducing effect of 10 μg/mL CA6-ErEVs was comparable to or greater than that of the positive control, Arb. Tyrosinase activity is the rate-limiting step during melanogenesis (Fernandez‐Julia et al. 2021; Schlessinger et al. 2025). Tyrosinase initiates melanin production by oxidizing tyrosine to L-DOPA and then to dopaquinone. In B16F10 cells, α-MSH enhanced cellular tyrosinase activity (Fig. 4c). Conversely, treatment with CA6-ErEVs (1 and 10 μg/mL) or Arb suppressed this effect. Notably, 10 μg/mL CA6-ErEVs markedly reduced tyrosinase activity to levels comparable to the untreated control, indicating antimelanogenic activity of CA6-ErEVs in murine melanoma cells. In contrast, LpEVs (*Lactobacillus paracasei*-derived EVs known for their strong anti-inflammatory and antioxidant activities (Lee et al. 2023)) increased melanin content (Supplementary Figure S2), demonstrating the strain-specific antimelanogenic activity of CA6-ErEVs.

To determine whether CA6-ErEVs exert antimelanogenic activity in primary human epidermal melanocytes (HEMs), we first assessed their cytotoxicity in moderately pigmented HEMs. Treatment with concentrations up to 100 μg/mL CA6-ErEVs did not reduce cell viability; rather, a slight increase in viability was observed (Fig. 4d). Under non-cytotoxic conditions, CA6-ErEVs treatment reduced melanin content and suppressed cellular tyrosinase activity in a dose-dependent manner, exhibiting greater inhibitory effects than Arb (Fig. 4e-f). These findings confirm effective antimelanogenic activity of CA6-ErEVs in HEMs, consistent with murine B16F10 cells. Notably, CA6-ErEVs isolated from *E. rotai* CMTB-CA6 culture medium without *C. asiatica* supplementation exhibited skin-whitening activity, similar to *C. asiatica* extracts with known antimelanogenic effects.

### CA6-ErEVs suppress melanogenesis via PDE activity, cAMP-MITF modulation and ROS reduction

To investigate the molecular mechanisms underlying the antimelanogenic properties of CA6-ErEVs, we examined their effects on intracellular signaling pathways involved in melanin synthesis. Melanogenesis is primarily regulated by the α-MSH-melanocortin 1 receptor (MC1R) signaling pathway through cAMP-dependent signaling (D’Mello et al. 2016; Hida et al. 2020). Additionally, ROS and hydrogen peroxide upregulate L-tyrosine synthesis, the precursor to melanin, contributing to skin darkening (Schallreuter et al. 2004; Snyman et al. 2024; Ahuja et al. 2025). Therefore, we assessed the effects of CA6-ErEVs on these key upstream regulators and their downstream effectors.

**Fig. 5:**
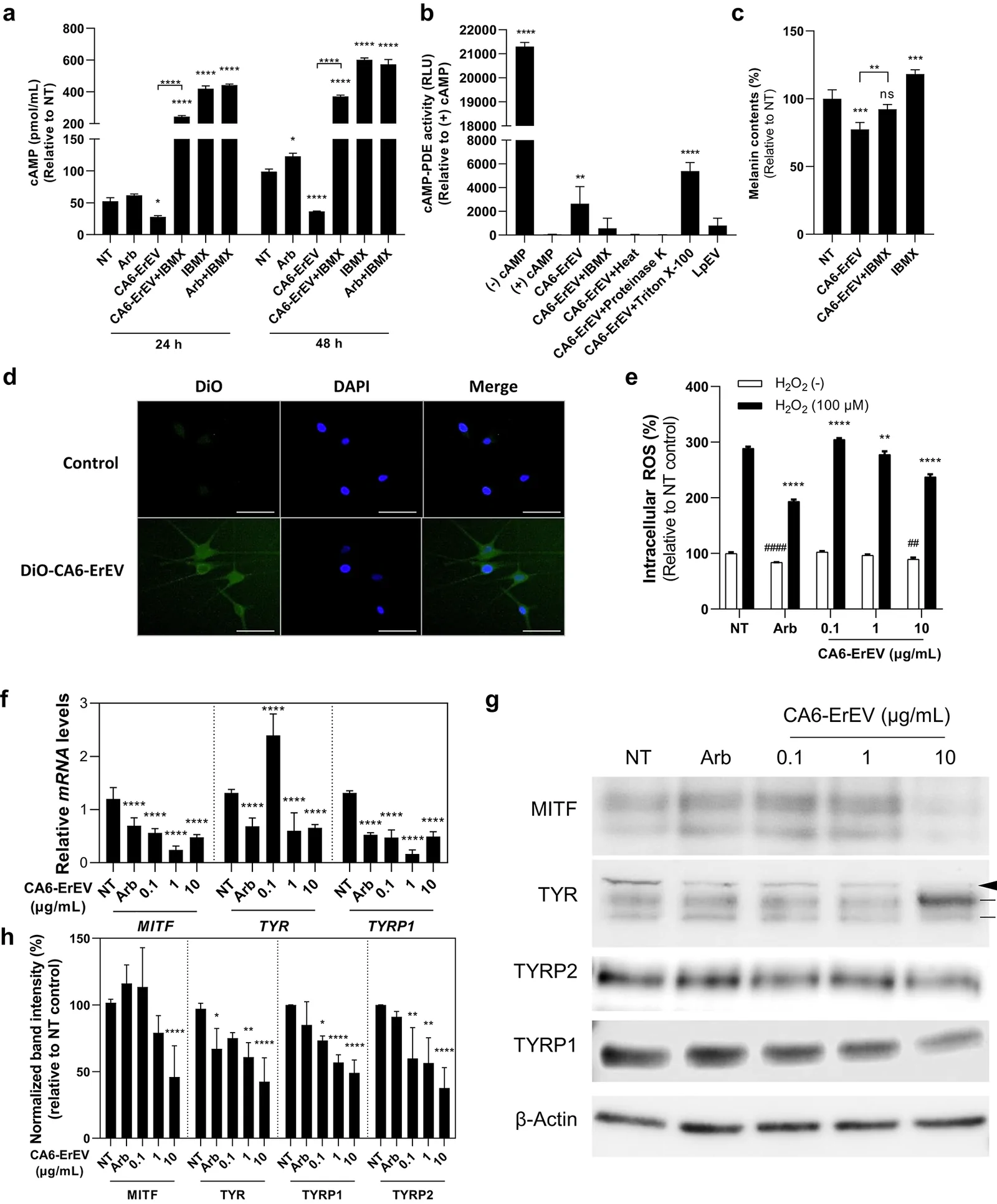
CA6-ErEVs suppress melanogenesis via cAMP-MITF modulation and ROS reduction. **a** Cyclic adenosine monophosphate (cAMP) levels were measured in the conditioned media of HEMs after treatment with CA6-ErEVs (0.1, 1, or 10 μg/mL), arbutin (Arb; 100 μg/mL), or 3-isobutyl-1-methylxanthine (IBMX; 100 nM), with IBMX serving as a phosphodiesterase (PDE) inhibitor. **b** PDE activity was initially analyzed in intact CA6-ErEVs and LpEVs, with LpEVs serving as the bacterial EV control. In separate experiments, PDE activity was measured after CA6-ErEVs were treated with IBMX (100 nM), heat (100 °C), proteinase K (50 μg/mL), or Triton X-100 (1%). **c** Melanin contents were quantified using melanin assay in HEMs treated with CA6-ErEVs (10 μg/mL) and IBMX (100 nM). Melanin content was normalized to the total protein content of each corresponding sample to account for differences in cell density. **d** The HEMs were treated with DiO (fluorescent membrane dye; green)-labeled CA6-ErEVs for 1.5 h, after which unabsorbed DiO-labeled CA6-ErEVs were washed away. After an additional 1.5 h incubation, the cell images were taken from fixed cells under a fluorescence microscope. Scale bar, 50 μm. **e** Intracellular ROS levels were determined in 100 μM H₂O₂-treated HEMs after CA6-ErEVs treatment. **f** Gene expression of melanogenesis-related genes (*MITF*, *TYR*, and *TYRP1*) was quantified using quantitative reverse transcription polymerase chain reaction (qRT-PCR) analysis, with *18S rRNA* used for normalization. **g**, **h** Western blot analysis was performed for melanogenic proteins (MITF, TYR, TYRP1, TYRP2), showing band intensity (**g**) and quantification (**h**) after CA6-ErEVs treatment. The TYR antibody detected multiple tyrosinase bands with distinct molecular weights. Relative protein levels were normalized to β-actin and quantified using densitometric analysis. The data are presented as mean ± SD (n = 4 for a; n = 3 for b, c; n = 4 for d, f). One-way ANOVA; * *p* < 0.05, ** *p* < 0.01, *** *p* < 0.001, **** *p* < 0.0001. Arbutin (Arb; 100 μg/mL), a standardized reference agent for melanogenesis inhibition, was used as the positive control. NT, no treatment; HEM, human epidermal melanocyte; ROS, reactive oxygen species; MITF, microphthalmia-associated transcription factor; TYR, tyrosinase; TYRP1, tyrosinase-related protein 1; TYRP2, tyrosinase-related protein 2

We measured intracellular cAMP levels in HEMs following CA6-ErEVs treatment. CA6-ErEVs (10 μg/mL) significantly reduced cAMP levels at 24 h and 48 h compared with the untreated control (Fig. 5a), suggesting suppression of early melanogenic signaling. To investigate the involvement of phosphodiesterase (PDE) activity, which regulates intracellular cAMP levels, cells were treated with the PDE inhibitor 3-isobutyl-1-methylxanthine (IBMX). Co-treatment with IBMX reversed the CA6-ErEV-induced reduction in intracellular cAMP levels, resulting in cAMP levels comparable to those observed with IBMX treatment alone. To determine whether CA6-ErEVs possess PDE activity, we directly measured PDE activity associated with CA6-ErEVs. CA6-ErEVs exhibited PDE activity consistent with cAMP degradation, whereas LpEVs showed no such activity (Fig. 5b). Pretreatment of CA6-ErEVs with IBMX (100 nM) significantly reduced their PDE activity. Similarly, heat treatment (100 °C) and proteinase K treatment (50 μg/mL) abolished PDE activity, whereas disruption of the vesicle membrane with Triton X-100 (1%) did not reduce PDE activity relative to untreated CA6-ErEVs (Fig. 5b). Furthermore, CA6-ErEVs (10 μg/mL) significantly reduced melanin content, whereas IBMX-pretreated CA6-ErEVs failed to significantly reduce melanin content compared with the untreated control (Fig. 5c). These findings suggest that CA6-ErEVs suppress melanogenesis, at least in part, through a PDE-dependent reduction in intracellular cAMP levels. To determine whether CA6-ErEVs are internalized by target cells and may thereby influence intracellular signaling, we performed an extracellular vesicle uptake assay using green fluorescent DiO-labeled CA6-ErEVs (10 μg/mL). Following incubation with HEMs, confocal laser scanning microscopy revealed clear cytoplasmic localization of green fluorescence surrounding the DAPI-stained nuclei, whereas control cells showed negligible background fluorescence (Fig. 5d). This observations demonstrate the cellular uptake of CA6-ErEVs by HEMs and support their potential to modulate intracellular signaling pathways, including cAMP-associated melanogenic signaling.

Following the evaluation of cAMP/PDE signaling, we next examined ROS as another upstream regulator of melanogenesis. Since CA6-ErEVs exhibited antioxidant activity in vitro and in HaCaT cells (Fig. 2), we assessed their effects on H₂O₂-induced intracellular ROS accumulation in HEMs. CA6-ErEVs treatment (1 and 10 μg/mL) significantly reduced intracellular ROS levels, as did Arb (Fig. 5e). Given that oxidative stress promotes melanogenesis, the ROS-scavenging activity of CA6-ErEVs provides an additional mechanism for their antimelanogenic effects.

Consistent with the suppression of upstream cAMP and ROS signaling, CA6-ErEVs treatment reduced the transcript levels of microphthalmia-associated transcription factor (*MITF*), tyrosinase (*TYR*), and tyrosinase-related protein 1 (*TYRP1*) (Fig. 5f). Correspondingly, the protein levels of MITF and its downstream targets, TYR, TYRP1, and TYRP2 were decreased following CA6-ErEVs treatment (Fig. 5g–h). Despite the overall reduction in total TYR protein levels, western blot analysis revealed multiple TYR-immunoreactive bands (Fig. 5g–h). Compared with the untreated control, treatment with CA6-ErEVs at 10 μg/mL decreased the intensity of the highest-molecular-weight TYR band, while increasing the intensities of lower-molecular-weight bands (Fig. 5g). These findings indicate that CA6-ErEVs reduced total TYR protein levels while altering the relative distribution of TYR-immunoreactive forms. Collectively, these results suggest that CA6-ErEVs inhibit melanogenesis by suppressing both cAMP-MITF signaling and ROS-mediated pathways.

### CA6-ErEVs suppress inflammation-induced melanogenesis triggered by IL-33

**Fig. 6:**
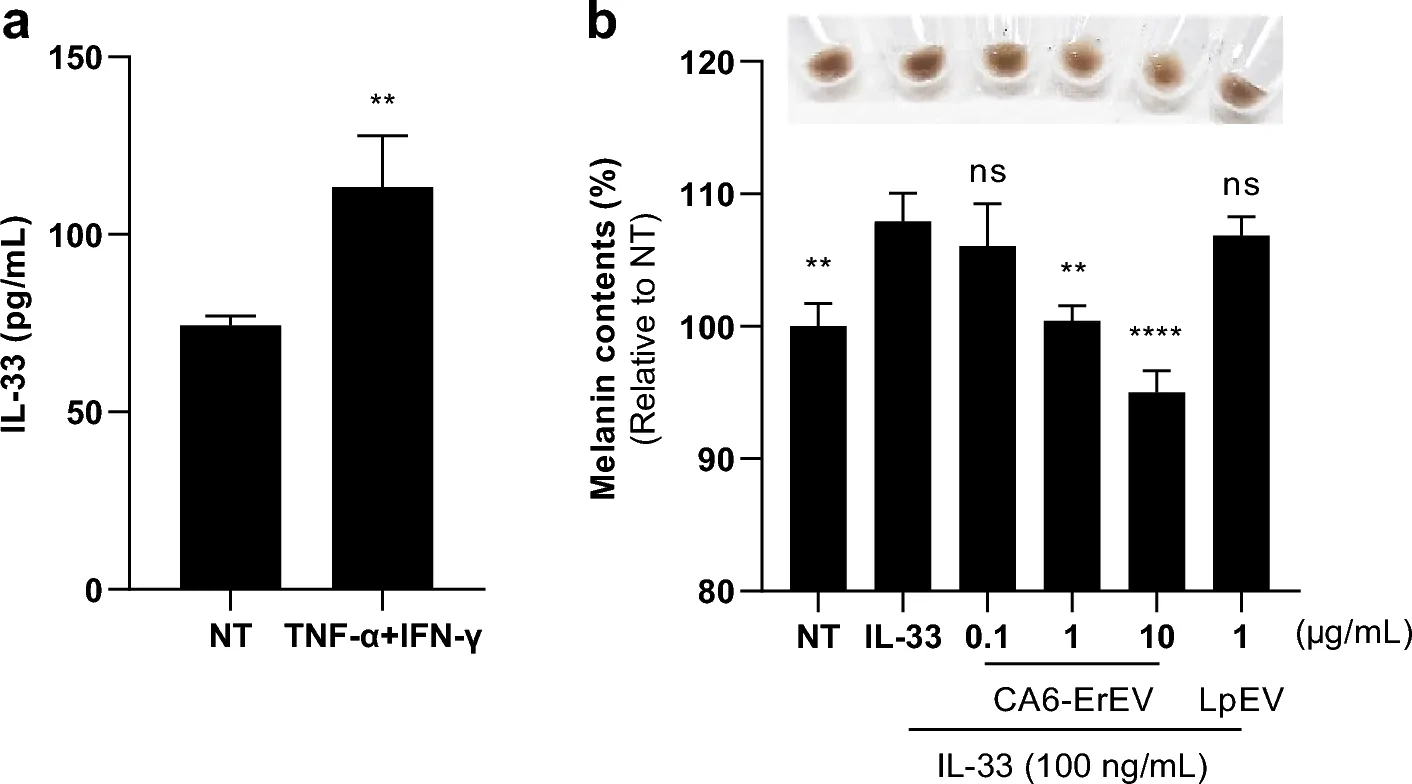
CA6-ErEVs attenuate IL-33-driven melanogenesis. **a** HaCaT cells were stimulated with TNF-α (40 ng/mL) and interferon-gamma (IFN-γ) (20 ng/mL), and IL-33 expression was quantified using a specific ELISA kit. **b** IL-33-stimulated HEMs were treated with CA6-ErEVs (0.1, 1, 10 μg/mL) or LpEVs (1 μg/mL), lysed with 2 M NaOH, and melanin content was determined by measuring absorbance at 405 nm using a microplate reader. The resulting values were normalized to the total protein content of each sample. Pellet of melanocytes prior to lysis was visualized. One-way ANOVA was performed. For panel (**a**), samples were compared to NT control. For panel (**b**), samples were compared to IL-33 alone. ** *p* < 0.01, **** *p* < 0.0001. NT, no treatment; HEM, human epidermal melanocyte

In addition to UVR-induced melanogenesis, inflammation-induced melanogenesis frequently occurs in response to inflammatory skin disorders, infection, and tissue injury (Fu et al. 2020; Hossain et al. 2021), leading to post-inflammatory hyperpigmentation. Among cytokines mediating inflammation-induced melanogenesis, IL-33 acts as a key alarm (Zhou et al. 2014). To evaluate IL-33 induction by inflammatory stimuli, we treated HaCaT keratinocytes with TNF-α (40 ng/mL) and interferon-gamma (20 ng/mL). Consistent with previous reports (Savinko et al. 2012; Balato et al. 2014), combined stimulation significantly increased IL-33 protein expression in HaCaT cells (Fig. 6a), recapitulating IL-33 release under inflammatory skin conditions. Direct IL-33 treatment on HEMs increased melanin content compared with that of the untreated control, however, CA6-ErEVs inhibited IL-33-induced melanin production in a dose-dependent manner, whereas LpEVs showed no effect (Fig. 6b).

## Discussion

In this study, we isolated and characterized EVs from *E. rotai* CMTB-CA6−a strain originally isolated from *C. asiatica*, known for its wound-healing and skin-brightening properties−and investigated their effects on human skin cells. CA6-ErEVs exhibit multifunctional bioactivities, including anti-inflammatory, antioxidant, and anti-melanogenic effects. Notably, this study provides the first evidence that LAB-derived EVs suppress melanogenesis via EV-associated PDE activity–mediated cAMP reduction, thereby expanding the functional repertoire of microbial EVs.

*E. rotai* CMTB-CA6 is a Gram-positive LAB strain originally isolated from the leaves of C. asiatica. Our previous genomic analysis revealed no primary virulence factors or acquired antibiotic resistance determinants but identified diverse catalytic and metabolic genes (Kim et al. 2024). Although a specific physiological relationship with *C. asiatica* has not been established, CMTB-CA6 attenuated ROS- and TNF-α-induced cellular stress. Because oxidative stress is an important regulator of melanogenesis, this demonstrated antioxidant activity provided the primary rationale for investigating the anti-melanogenic effects of CA6-ErEVs. The PDE activity identified in this study provides additional mechanistic support for their regulation of melanogenic signaling.

A primary objective was to examine whether CA6-ErEVs provide functional advantages over parental *E. rotai* CMTB-CA6 lysates. As demonstrated with LpEVs, which showed superior collagen synthesis activity compared to parental lysates (Lee et al. 2023), CA6-ErEVs exhibited enhanced biological efficacy across skin-related functions, particularly antioxidant activity. This superior potency aligns with reports that bacterial EVs outperform lysates through selective cargo loading, molecular stabilization, and efficient delivery (Amabebe et al. 2024). Given the central role of oxidative stress in inflammatory matrix degradation and pigmentation, CA6-ErEVs represent promising antioxidant agents for dermatological and cosmetic applications.

The strain-specific anti-melanogenic activity of CA6-ErEVs raises the possibility that adaptation to the *C. asiatica*-associated ecological niche may have influenced their functional properties. Plant-derived EVs can mediate inter-kingdom communication by delivering functional cargo, including small RNAs and bioactive metabolites, to interacting microorganisms (He et al. 2021; Wang et al. 2024; Li et al. 2024; Ayala-García et al. 2025; Ragni 2025). Accordingly, it is biologically plausible that *C. asiatica*-derived EVs could influence the physiology or metabolism of associated bacteria through cargo transfer. However, there is currently no direct evidence that *C. asiatica*-derived EVs are taken up by CMTB-CA6 or that anti-melanogenic or other specific biological activities are transferred from the plant to the bacterium. Therefore, EV-mediated plant–bacterium communication remains an ecological hypothesis that requires direct experimental validation.

The PDE activity associated with CA6-ErEVs provides a potential mechanistic basis for their regulation of melanogenic signaling. PDEs regulate intracellular cAMP levels through enzymatic hydrolysis and thereby control diverse cAMP-dependent cellular responses (Bolger et al. 1993; Houslay and Adams 2003). In this context, EV-associated PDE activity suggests that bacterial EVs may influence host signaling through the presentation or delivery of catalytic enzymes. Nevertheless, the precise localization of PDE within CA6-ErEVs and the mechanism by which it gains access to intracellular cAMP remain unresolved. Although the observed cellular uptake of CA6-ErEVs provides a possible route, direct intracellular delivery and activity of the responsible PDE enzymes require further validation.

Most previous PDE-related studies have focused on the biological effects of PDE inhibitors (Bondarev et al. 2022), whereas the delivery of catalytically active PDEs by bacterial EVs remains poorly characterized. Phosphodiesterase-related enzymes have been reported in microbial EVs, including glycerophosphoryl-diester phosphodiesterase in Streptomyces lividans (Schrempf and Merling 2015) and CD73, which hydrolyzes AMP rather than cAMP (Winzer et al. 2024). These reports provide a relevant precedent for EV-associated phosphodiesterase activity but do not establish direct cAMP hydrolysis by bacterial EVs. Therefore, CA6-ErEV-associated PDE activity may represent an underexplored mechanism of host–microbial communication, although its molecular identity, vesicular localization, and intracellular action require further investigation.

As a downstream effect of cAMP suppression, CA6-ErEVs coordinately downregulated MITF and melanogenic enzymes. CA6-ErEVs also reduced the higher-molecular-weight TYR band, accompanied by an increase in lower-molecular-weight TYR-immunoreactive bands (Fig. 5g). Because glycosylation is important for TYR folding, ER-to-Golgi trafficking, and enzymatic activation (Ando et al. 2007; Mikami et al. 2013), this altered banding pattern may reflect changes in post-translational processing. A similar pattern has been reported under conditions of defective mannose trimming, which leads to ER retention of immature tyrosinase and subsequent degradation (Wang and Androlewicz 2000). However, neither the glycosylation status nor the maturation states of the individual TYR bands were directly determined in this study. Therefore, whether the observed changes are related to altered glycosylation, intracellular trafficking, or other processing events remains unclear. Further studies using glycosylation inhibitors, glycosidase treatments, and intracellular trafficking markers will be required to clarify the underlying mechanism. Thus, while the present findings support suppression of the cAMP-MITF pathway, a potential effect of CA6-ErEVs on TYR maturation remains a hypothesis requiring further validation.

Beyond the α-MSH–cAMP axis, CA6-ErEVs' ability to attenuate inflammation-driven pigmentation pathways highlights their therapeutic versatility for conditions such as post-inflammatory hyperpigmentation, where cytokine-mediated melanogenesis predominates over hormonal triggers. This broad-spectrum efficacy stems from their unique capacity to decouple antioxidant/anti-inflammatory properties from whitening activity—a functional divergence not observed in other LAB-derived EVs. While shared bioactivities may partially attenuate inflammatory signaling, CA6-ErEVs exert robust suppression of melanogenesis, highlighting strain-specific functional specialization likely shaped by adaptation to plant-associated niches. Notably, their plant-independent mechanism distinguishes CA6-ErEVs from *C. asiatica*–derived triterpenoids, such as madecassoside, which require MC1R engagement (Seo et al. 2025). This microbial origin enables GMP-compatible, scalable production with reduced batch-to-batch variability compared to phytochemicals. Furthermore, the intrinsic skin penetration and cellular delivery properties of EVs support efficient topical bioavailability (Yang et al. 2021; Lee et al. 2023; Ferroni et al. 2025).

Overall, the present study demonstrates the beneficial effects of CA6-ErEVs on multiple skin-related cellular responses and provides a foundation for their potential dermatological application. From a translational perspective, future studies should build on these findings through comprehensive characterization of EV components, including potentially immunostimulatory molecules such as LTA, together with appropriate immunogenicity, skin irritation, sensitization, and in vivo safety assessments. Such studies will help define the safety profile of CA6-ErEVs and support their further development as skin-active biological materials.

## Conclusions

CA6-ErEVs exhibited antioxidant activity and exerted anti-inflammatory and matrix-protective effects in TNF-α-treated HDFs. In melanocytes, CA6-ErEVs reduced melanin production by modulating cAMP-MITF and ROS-related signaling, with EV-associated PDE activity contributing, at least in part, to these effects. Collectively, these findings support CA6-ErEVs as a promising LAB-derived bioactive candidate, although further validation in physiologically relevant models is required.

## Methods

### Cell culture

HDFs were obtained from ATCC (Manassas, VA, USA). Neonatal HEM and HaCaT keratinocytes were purchased from Cytion (Eppelheim, Germany). The murine melanoma cell line B16F10 was obtained from the Korean Cell Line Bank (Seoul, Republic of Korea). Cells were cultured under standard conditions using the recommended media and supplements and maintained at 37 °C in 5% CO₂. Further details on cell culture are provided in the Supplementary Information.

### Preparation of *E. rotai*-derived CA6-ErEVs

*Enterococcus rotai* CMTB-CA6 strain used for extracellular vesicle (EV) production was isolated from *Centella asiatica* (Kim et al. 2024). *E. rotai* CMTB-CA6-derived extracellular vesicles (CA6-ErEVs) were prepared as follows: *E. rotai* CMTB-CA6 was inoculated into the MRS medium (BD Biosciences, Sparks, MD, USA) and cultured at 37 °C with shaking at 200 rpm for 20 h until the optical density at 600 nm reached 0.8–1.0. The primary culture was then inoculated into YPG medium (1% yeast extract, 1.5% soy peptone, and 3% glucose (all from LPS Solution, Daejeon, Republic of Korea), pH 8.0) developed by CHA Meditech (Seongnam, Republic of Korea) for secondary culture. For large-scale culture, 100 L of YPG medium was prepared in a bioreactor and inoculated with the secondary culture. The fermenter was set to 37 °C, 150 rpm, and an air flow rate of 200 L/min, and the culture was incubated for an additional 20 h. Cultured bacterial cells were harvested and continuously centrifuged at 15 600 rpm with a flow rate of 1.8 L/min. The resulting supernatant was subsequently filtered through a 0.5 μm Polysep TM II cartridge (Merck Millipore, Burlington, MA, USA). The filtered supernatant was concentrated to a final volume of less than 1 L using a 100 kDa Pellicon 2 Cassette filter membrane (tangential flow filtration (TFF); Merck Millipore). The concentrated filtrate was then passed through a 0.2 μm demicap propor HC filter (Parker Hannifin Co., Oxnard, CA, USA) to obtain CA6-ErEVs. The size distribution and particle concentration of isolated EVs were determined using a ZetaView nanoparticle tracking analysis system (NTA; Particle Metrix GmbH, Inning am Ammersee, Germany). The protein concentration was quantified using the Pierce BCA Protein Assay Kit (Thermo Fisher Scientific). The morphology and size of EVs were observed using a Hitachi HT7800 transmission electron microscope (TEM; Hitachi High-Tech, Tokyo, Japan). The entire 100 L large-scale production and isolation process was performed in three independent batches, demonstrating consistent physical properties and yields of CA6-ErEVs across all batches. For efficacy assays, 0.1 μg/mL was selected as the reference concentration based on our previous dose-ranging study, in which LpEVs exhibited robust biological activity at this concentration (Lee et al. 2023).

### Cell viability assays

Cell viability was assessed using the EZ-Cytox assay kit (DoGenBio, Seoul, Korea). HDFs (1 × 10^4^ cells/well), B16F10 (5 × 10^5^ cells/well), and HEMs (4 × 10^5^ cells/well) were seeded and treated with CA6-ErEVs under inflammatory (TNF-α) or melanogenic (α-MSH) conditions, as indicated. Absorbance was measured at 450 nm at multiple time points. Detailed assay protocols are provided in the Supplementary Information.

### Analysis of antioxidant activity

The antioxidant capacity of probiotic extracellular vesicles (EVs) was assessed using the oxygen radical absorbance capacity (ORAC) method. A reaction mixture of 80 nM sodium fluorescein (Sigma-Aldrich, USA) and 75 mM phosphate buffer (pH 7.4) was prepared. This mixture was combined with 25 μL of either CA6-ErEVs, *E. rotai* CMTB-CA6 lysates, or vitamin C (Sigma-Aldrich, USA) at different concentrations. Subsequently, 50 μL of 75 mM AAPH (2,2′-Azobis(2-methylpropionamidine) dihydrochloride; Sigma-Aldrich, USA) was added to initiate the reaction. The fluorescence was measured at 485 nm excitation/528 nm emission every min for 60 min at 37 °C. As a negative control, the experiment was conducted by adding only the solvent used for sample dilution. Probiotic EVs and lysates were dissolved in PBS at varying concentrations. The area under the curve (AUC) of the fluorescence decay curve was calculated using the following mathematical formula: AUC = 1 + *f*_*1*_*/f*_*0*_ + … + *f*_*i*_*/f*_*0*_ + … + *f*_*60*_*/f*_*0*_ (where *f*_*0*_ represents the fluorescence value at 0 min, and *f*_*i*_ represents the fluorescence value at *i* min). The final antioxidant capacity was determined by calculating the net AUC, which was determined by subtracting the AUC value of the negative control from the measured AUC value. The net AUC was calculated by subtracting the AUC of the negative control from that of the sample. Finally, the antioxidant capacity was quantitatively expressed as Trolox equivalents (μmol TE/g) using a Trolox standard curve.

Intracellular ROS levels were determined using the CM-H_2_DCFDA assay (Invitrogen, USA) according to the manufacturer’s instructions. Briefly, HaCaT cells or HEMs were pre-treated with CM-H_2_DCFDA (1 μM) and CA6-ErEVs at different concentrations (0, 0.1, 1, 10, 100 μg/mL) for 24 h. Subsequently, the cells were challenged with 100 μM H_2_O_2_ for 1 h. Following two washes with PBS, DCF fluorescence was measured at excitation/emission wavelengths of 485/530 nm.

### Enzyme-linked immunosorbent assay (ELISA)

The levels of pro-collagen I alpha 1, MMP-1, IL-6, IL-8, and cAMP in the cultured media were quantified following treatment with TNF-α (20 ng/mL) or α-MSH (100 nM) in combination with CA6-ErEVs. Specific ELISA kits for pro-collagen I alpha 1, MMP-1, IL-6, IL-8, and cAMP (all from R&D Systems, USA) were employed for each assay following the manufacturer’s instructions. The detection limits of each ELISA kit were as follows: pro-collagen I alpha 1 (31.2–2000 pg/mL), MMP-1 (62.5–4000 pg/mL), IL-6 (9.4–600 pg/mL), IL-8 (31.2–2000 pg/mL), cAMP (3.75–240 pmol/mL). The levels of IL-33 in the cultured media were quantified following treatment with TNF-α (40 ng/mL) and IFN-g (20 ng/mL). Specific ELISA kits for IL-33 (R&D Systems, USA) were used according to the manufacturer’s instructions. The absorbance was read at 450 nm using a microplate reader, and the concentration of IL-33 was determined based on a standard curve.

### Melanin content and cellular tyrosinase activity assay

Melanin content was quantified spectrophotometrically following alkaline lysis of treated cells. Cellular tyrosinase activity was determined by monitoring dopachrome formation from L-DOPA and normalized to total protein content. The methodology is detailed in the Supplementary Information.

### Western blot analysis

Total cellular proteins were extracted, separated using sodium dodecyl sulfate–polyacrylamide gel electrophoresis, and transferred to polyvinylidene fluoride membranes. Membranes were probed with antibodies against melanogenesis-related proteins and detected using enhanced chemiluminescence. Band intensities were quantified using ImageJ software (Fiji v1.54p; NIH, Bethesda, MD, USA). Detailed protocols and antibody information are available in the Supplementary Information.

### Quantitative reverse transcription-polymerase chain reaction (qRT-PCR)

Total RNA was isolated using a commercial kit (Zymo Research, USA), reverse-transcribed (TOYOBO, Japan), and subjected to quantitative PCR using SYBR Green chemistry (Roche, Switzerland). Relative gene expression levels were calculated using the 2^−ΔΔCt^ method and normalized to *18S* rRNA expression. Detailed protocols and primer sequences are provided in the Supplementary Information.

### PDE activity assay

PDE activity associated with intact CA6-ErEVs was measured using a PDE-Glo™ Phosphodiesterase Assay Kit (Promega, Madison, WI, USA), according to the manufacturer’s instructions. The methodology is detailed in the Supplementary Information.

### Statistical analyses

All data are presented as the mean ± standard deviation of at least three independent experiments. Statistical comparisons were performed using a one-way analysis of variance, followed by Tukey’s post hoc test (GraphPad Prism version 8.0.1, GraphPad Software Inc. USA). A *p*-value < 0.05 was considered statistically significant.

## Supplementary information

Supplementary Material 1.

Supplementary Material 2.

## Data Availability

The original contributions presented in the study are included in the article/Supplementary Information. The data that support the findings of this study are available from the corresponding author upon reasonable request.

## Abbreviations

CA6-ErEVs: Extracellular vesicles derived from *Enterococcus rotai* CMTB-CA6
NTA: Nanoparticle tracking analysis
ELISA: Enzyme-linked immunosorbent assay
HDFs: Human dermal fibroblasts
TNF-α: Tumor necrosis factor-α
HEMs: Human epidermal melanocytes
ROS: Reactive oxygen species
α-MSH: α-Melanocyte–stimulating hormone
MC1R: Melanocortin 1 receptor
AC: Adenylyl cyclase
cAMP: Cyclic adenosine monophosphate
PKA: Protein kinase A
CREB: cAMP response element–binding protein
MITF: Microphthalmia-associated transcription factor
TYR: Tyrosinase
TYRP: Tyrosinase-related protein
PAH: Phenylalanine hydroxylase
IL-33: Interleukin-33
ST2: Suppression of tumorigenicity 2
