# Supplementary material for: Extracellular vesicles derived from the lactic acid bacterium Enterococcus rotai suppress melanogenesis by modulating the cAMP-MITF pathway
Source: Bioresour Bioprocess. 2026 Sep 12;13(1):134. doi: 10.1186/s40643-026-01121-5 (PMC13570877; doi:10.1186/s40643-026-01121-5)
Supplement: Supplementary file 1 [file 40643_2026_1121_MOESM2_ESM.docx]

**Supplementary Information**

Extracellular vesicles derived from the lactic acid bacterium *Enterococcus rotai* suppress melanogenesis by modulating the cAMP-MITF pathway

Yunsik Kim, Jin Hee Lee, Eun-Gyung Cho

**Supplementary materials and methods**

**Cell Culture**

Human dermal fibroblasts (HDFs) were purchased from ATCC (Manassas, VA, USA). Moderately pigmented neonatal human primary epidermal melanocytes (HEMs) and spontaneously immortalized HaCaT cells were purchased from Cytion (Eppelheim, Germany). The murine melanoma cell line B16F10 was obtained from the Korean Cell Line Bank (Seoul, Republic of Korea). HDFs, HaCaT cells, and B16F10 cells were cultured in Dulbecco’s modified Eagle medium (DMEM; WELGENE, Republic of Korea) supplemented with 10% fetal bovine serum (FBS; Thermo Fisher Scientific, Waltham, MA, USA). HEMs were cultured in Medium 254 (Thermo Fisher Scientific) containing human melanocyte growth supplement (HMGS; Thermo Fisher Scientific). All culture media contained 100 units/mL penicillin–streptomycin (Thermo Fisher Scientific), and all cells were cultured at 37°C in 5% CO_2_.

**Cell Viability Assay**

To measure cell viability, HDFs (1 × 10^4^ per well) were seeded in a 48-well plate and cultured for 24 h in a growth medium. To establish inflammatory conditions, cells were incubated under serum-free conditions for 6 h. Cells were pretreated with TNF-α (20 ng/mL; R&D Systems, USA) for 24 h, followed treatment with each sample at various concentrations for an additional 24 h in the continued presence of TNF-α. For the viability assay, 20 μL of EZ-Cytox (DoGenBio, Republic of Korea) was added to each well, incubated for 1 h, and the absorbance was measured at 450 nm. For B16F10 cells (5 × 10^5^ per well), cells were cultured in a 96-well plate for 24 h. Then, the cells were co-treated with α-MSH (100 nM; Sigma-Aldrich, USA) and either arbutin (100 μg/mL; Sigma-Aldrich, USA) or CA6-ErEVs at various concentrations for 48 h. Cell Counting Kit-8 (CCK-8; Dojindo, Republic of Korea) was added to each well, incubated for 1 h, and absorbance was measured at 450 nm. For HEMs (4 × 10^5^ per well), cells were cultured in a 6-well plate for 24 h. Then, the cells were subsequently treated with arbutin (100 μg/mL; Sigma-Aldrich, USA) or CA6-ErEVs at various concentrations. This treatment was repeated daily for three consecutive days. EZ-cytox was then added to each well, incubated for 1 h, and absorbance was measured at 450 nm.

**Melanin contents and cellular tyrosinase activity assay**

B16F10 cells (5 × 10^5^ cells/well) and HEMn-MP cells (4 × 10^5^ cells/well) were seeded in 6-well plates, and incubated for 24 h. B16F10 cells were co-treated with α−MSH (100 nM) and CA6-ErEVs at various concentrations for 48 h. HEMn-MP cells were treated with CA6-ErEVs once daily for three consecutive days. The cells were then washed with PBS and lysed with 2 M NaOH. The amount of synthesized melanin was determined by measuring the absorbance of the cell lysates at 405 nm using a microplate reader.

Cellular tyrosinase activity was measured in B16F10 cells and HEMn-MP cells. Following the same treatment protocol as the melanin content assay, cells were washed twice with PBS and lysed with RIPA buffer (Sigma-Aldrich, USA). The protein concentration of the lysates was quantified using the Pierce BCA protein assay kit (Thermo Fisher Scientific, USA). A total of 200 μL of the cell lysate was incubated with 2 mM L-DOPA at 37°C for 1 h. The formation of dopachrome was monitored by measuring the absorbance at 450 nm. Tyrosinase activity was normalized to the total protein concentration.

**Measurement of cAMP levels**

To evaluate the effect of CA6-ErEVs on cAMP levels, human epidermal melanocytes (HEMn) were seeded in 6-well plates at a density of 4 × 10⁵ cells/well and cultured at 37°C in 5% CO₂. Cells were treated with CA6-ErEVs (10 μg/mL), arbutin (100 μg/mL), or IBMX (3-isobutyl-1-methylxanthine; 100 μM; Sigma-Aldrich, St. Louis, MO, USA), a broad-spectrum PDE inhibitor, every 24 h for three consecutive days. Conditioned media were collected at 24, 48, and 72 hours, and stored at ≤ −80°C until analysis. The cAMP concentrations were quantified using the cAMP Parameter Assay Kit (R&D Systems, Minneapolis, MN, USA) according to the manufacturer's instructions. Briefly, the collected conditioned media were diluted with Calibrator Diluent. A seven-point standard curve was prepared by serial dilution of the cAMP standard stock solution in Calibrator Diluent. Primary antibody solution was added to each well and incubated for 1h, followed by the addition of 50 μL of cAMP conjugate and 100 μL of standard, control, or sample. The plate was sealed and incubated for 2 h at room temperature. After incubation, the wells were washed four times, and 200 μL of Substrate Solution was added to each well. The plate was incubated for 30 minutes at room temperature protected from light. The reaction was terminated by the addition of 100 μL of Stop Solution, and the absorbance was measured at 450 nm (with correction at 570 nm) using a microplate reader within 30 min. Because the assay is based on a competitive immunoassay, the color intensity is inversely proportional to the cAMP concentration. cAMP concentrations were calculated using a four-parameter logistic (4-PL) standard curve and expressed as pmol/mL.

**PDE activity assay**

PDE activity was assessed using a PDE-Glo^TM^ Phosphodiesterase Assay Kit (Promega, Madison, WI, USA) according to the manufacturer's instructions. Briefly, CA6-ErEVs were prepared, and the protein concentration was determined. To evaluate the characteristics of the PDE activity, CA6-ErEVs were pretreated with IBMX (0.5 mM), Triton X-100 (1%, v/v), proteinase K (50 μg/mL), or heat (100 °C for 5 min). Following proteinase K treatment, the enzyme was inactivated with a serine protease inhibitor, and the vesicles were washed using ExoQuick UltraPure (System Biosciences, Palo Alto, CA, USA) to remove residual proteinase K. Intact or pretreated CA6-ErEVs were then incubated with 2 μM cAMP for 60 min at 25 °C to initiate the PDE reaction. The reaction was terminated by the addition of PDE-Glo™ Termination Buffer. Subsequently, the PDE-Glo™ Detection Reagent, containing protein kinase A (PKA), was added, followed by the Kinase-Glo® Reagent to determine the amount of ATP remaining. Because PDE hydrolyzes cAMP, less PKA is activated, resulting in reduced ATP consumption. Consequently, more ATP remains available for the luciferase reaction, producing a luminescence signal that is directly proportional to the remaining ATP level and, therefore, to PDE activity.

**Fluorescence microscopy analysis of CA6-ErEV internalization**

To evaluate the cellular internalization of CA6-ErEVs, extracellular vesicles were labeled with the lipophilic fluorescent dye DiO (Invitrogen, Waltham, MA, USA). Briefly, 2 mL CA6-ErEVs were incubated with 4 µg/mL DiO for 1.5 h at room temperature in the dark, according to the manufacturer’s instructions. Free dye was removed using 10 kDa MWCO centrifugal filter units (Merck Millipore, Burlington, MA, USA) by ultrafiltration at 3,000 × g for 10 min. The samples were washed with phosphate-buffered saline (PBS) until the free dye was completely removed.

Human epidermal melanocytes (HEMs) were seeded on a chamber slide and cultured to an appropriate confluency. The cells were then treated with DiO-labeled CA6-ErEVs (50 µg/mL) for 1 h 30 min at 37°C. Following incubation, the cells were washed twice with PBS to remove unbound vesicles, and fixed with 4% paraformaldehyde (PFA) at room temperature for 15 min. After fixation, the cells were washed with PBS and mounted using a VECTASHIELD antifade mounting solution (containing 1.5 µg/mL DAPI as a nuclear counterstain; Vector Laboratories, Burlingame, CA, USA) with coverslips onto the chamber slide. The cellular uptake and intracellular fluorescence distribution of DiO-CA6-ErEVs were captured and observed using a fluorescence microscope (IX73 inverted microscope; Olympus, Shinjuku, Japan).

**Quantitative Reverse Transcription-Polymerase Chain Reaction (qRT-PCR)**

Total RNA was extracted from cells using an RNA isolation kit (Zymo research, USA) according to the manufacturer’s protocol. Total RNA concentration and purity were assessed using a nano drop spectrophotometer (IMPLEN Gmbh, Germany). Complementary DNA (cDNA) was synthesized from 1 μg of total RNA using a reverse transcription kit (TOYOBO, Japan). qRT-PCR was performed using a Lightcycler 96 real-time PCR system (Roche, Switzerland) with gene-specific primers and a SYBR green master mixture (Roche, Switzerland). The relative mRNA expression levels of target genes were calculated using the 2^－ΔΔ^Ct method, normalized to *18S rRNA* gene. Primers used for the amplification are listed at **Table 1**.

**Table 1. Gene specific primer sequence for qRT-PCR.**

| **Gene name** | **Forward primer (5’ - 3’)** | **Reverse primer (5’ - 3’)** |
| --- | --- | --- |
| ***MITF*** | **ATCCTGATGGACGACACCCTTT** | **AAGTGTGCTCCGTCTCTTCCAT** |
| ***TYR*** | **TAGTCCACTTACTGGGATAGCG** | **GAAGGAAGATAGGATCGTTGGC** |
| ***TYRP1*** | **GCAGTAGTTGGCGCTTTGTTAC** | **GATACTGATCAGTGAGGAGAGG** |
| ***18S rRNA*** | **CGGCTTTGGTGACTCTAGAT** | **GCGACTACCATCGAAAGTTG** |

**Western blot analysis**

HEMn-MP cells were harvested and lysis in PRO-PREP buffer (iNtRON biotechnology, Republic of Korea) with a protease and phosphatase inhibitor cocktail (Sigma-Aldrich, USA). Protein concentrations were measured using the BCA assay kit. Equal amounts of protein (40 μg per lane) were mixed with 5× SDS sample buffer (Biosesang, Republic of Korea) to the 10% SDS-PAGE gel and subsequently transferred with X 1 transfer buffer (LPS solution, United Kingdom) to a PVDF membrane (Merck, USA). The membranes were blocked in 5% skim milk (LPS solution, United Kingdom) in X1 TBST (LPS solution, United Kingdom) for 1 h at room temperature. The membranes were incubated overnight at 4℃ with target specific primary antibodies (MITF (Cell Signaling, USA), TYR (invitrogen, USA), TYRP1 (Cell Signaling, USA), TYRP2 (Novus Biologicals, USA)) followed by incubation with the appropriate HRP-conjugated secondary antibodies for 2 h at room temperature. Protein bands were detected using an enhanced chemiluminescence (ECL) detection kit (Thermo Fisher Scientific, USA) and LAS instrument (GE HealthCare, Sweden). The band intensity was quantified using image J program (https://imagej.net/).

**Purification of extracellular vesicles by OptiPrep density gradient ultracentrifugation**

CA6-ErEVs were further purified using discontinuous iodixanol density gradient ultracentrifugation. A discontinuous density gradient was prepared by sequentially layering 1.3 mL each of 50%, 30%, and 10% OptiPrep™ solutions (Axis-Shield, Oslo, Norway), diluted in 0.25 M sucrose, from the bottom to the top of an OptiSeal tube (1/2 in.; Beckman Coulter, Brea, CA, USA). One milliliter of the EV suspension was carefully loaded onto the top of the gradient, resulting in a total volume of 4.9 mL. Ultracentrifugation was performed using an NVT90 rotor (Beckman Coulter) at 200,000 × g for 2 h at 4°C. Following centrifugation, 0.45-mL fractions were sequentially collected from the top of the gradient, yielding approximately 10–11 fractions. The density of each fraction was determined by gravimetric analysis. Briefly, the mass of each 0.45-mL fraction was measured using an analytical balance, and the density was calculated by dividing the measured mass by the corresponding volume. The iodixanol concentration of each fraction was subsequently estimated using a standard calibration curve generated from OptiPrep standards (0–50%, diluted in 0.25 M sucrose) prepared and measured by the same gravimetric method. EVs were recovered in fraction #7, which corresponded to a buoyant density of 1.12–1.14 g/mL and an iodixanol concentration of approximately 20%, consistent with the reported buoyant density range of small extracellular vesicles isolated by iodixanol density gradient ultracentrifugation (Théry et al. 2006; Kowal et al. 2016). Fraction #7 (~0.45 mL) was diluted to a final volume of 5 mL with phosphate-buffered saline (PBS) and subjected to a washing ultracentrifugation at 150,000 × g for 2 h at 4°C to remove residual iodixanol. After careful aspiration of the supernatant, the EV pellet was resuspended in 200 μL of PBS and used for subsequent analyses

**Supplementary data**

**Supplementary Figure S1.** Comprehensive characterization and purity analysis of CA6-ErEVs. (a) Coomassie brilliant blue-stained SDS-PAGE gel showing protein profiles of UC-MSC-EVs, CA6-WL, CA6-ErEVs, and LpEVs (10 μg protein loaded per lane). Arrows indicate distinct differences in protein band patterns between CA6-WL and CA6-ErEVs. (b) DNA-to-RNA ratio analysis of CA6-WL and CA6-ErEVs. Nucleic acids were extracted using TRIzol, and the relative ratio of RNA to DNA was calculated based on their measured concentrations. (c) Optiprep density gradient ultracentrifugation image and fraction profile. Eleven fractions were collected from the top to the bottom, and their respective densities and Optiprep concentrations (%) are summarized in the table. Purified CA6-ErEVs were recovered and collected from fraction F7 for subsequent experiments. (d) Physical properties and particle size distribution of Optiprep density gradient-purified CA6-ErEVs determined using nanoparticle tracking analysis (NTA).

**Supplementary Figure S2.** Effects of LpEVs on melanogenesis in B16F10 cells. (a) B16F10 cells were treated with LpEVs (0.1, 1, 10 μg/mL) or arbutin (Arb, 100 μg/mL), and cell viability was determined using the CCK-8 assay. (b) α-MSH-stimulated (100 nM) B16F10 cells were treated with LpEVs or arbutin (Arb, 100 μg/mL, positive control), and melanin content was quantified by measuring absorbance at 405 nm after lysis with 2 M NaOH. Arb (100 μg/mL) was used as a standardized positive control for melanin inhibition. Data are mean ± SD (n = 6). One-way ANOVA; **** *p* < 0.0001. NT, no treatment; α-MSH, α-melanocyte-stimulating hormone

**Supplementary Figure S3.** Effects of CA6-ErEVs on melanogenesis-related gene expression in B16F10 cells. (a-c) mRNA expression of *tyrosinase* (*TYR*), *tyrosinase-related protein 1* (*TYRP1*), and *myosin Va* (*MYO5A*) was quantified by qRT-PCR in CA6-ErEVs-treated cells. LpEVs served as a comparative bacterial EV control to demonstrate the strain-specific anti-melanogenic activity of CA6-ErEVs. Relative expression levels were calculated using the 2^−ΔΔ^Ct method and normalized to *β-actin*. Data are mean ± SD (n = 3). One-way ANOVA; **** *p* < 0.0001.

10

15

25

35

40

55

180

100

70

kDa

**LpEV**

**CA6-ErEV**

**CA6-WL**

**MSC-EV**

**d**

**c**

**b**

**Supplement Figure S1.**

**a**

F11

F10

F9

F8

**F7**

F6

F5

F4

F3

F2

F1

1.33 × 10^12^ particles/mL

9.32 × 10^8^ particles/μg
Mean size: 166.34 nm
Mode size: 122.5 nm

**c**

**b**

**a**

**Supplement Figure S3.**

**b**

**a**

**Supplement Figure S2.**
